# Agricultural landscape and spatial distribution of *Toxoplasma gondii* in rural environment: an agent-based model

**DOI:** 10.1186/1476-072X-13-45

**Published:** 2014-10-28

**Authors:** Cécile Gotteland, Brent M McFerrin, Xiaopeng Zhao, Emmanuelle Gilot-Fromont, Maud Lélu

**Affiliations:** Université de Lyon, Université Lyon 1; CNRS, UMR5558, Laboratoire de Biométrie et Biologie Evolutive, Bâtiment Mendel, Université Lyon 1, 43 Bd du 11 novembre 1918, F-69622 Villeurbanne, France; Université de Reims Champagne-Ardenne, Laboratoire de Parasitologie-Mycologie, EA 3800, UFR de Médecine, SFR Cap Santé FED 4231, 51 rue Cognacq-Jay, F-51096 Reims, France; Department of Mechanical, Aerospace, and Biomedical Engineering, University of Tennessee, Knoxville, TN 37996 USA; National Institute for Mathematical and Biological Synthesis, University of Tennessee, Knoxville, TN 37996 USA; Université de Lyon, VetAgro-Sup Campus Vétérinaire, 1 Avenue Bourgelat, F-69280 Marcy l’Etoile, France; Division of Epidemiology and Community Health, School of Public Health, University of Minnesota, 1300 S. Second st. Suite 300, Minneapolis, MN 55454 USA

**Keywords:** *Toxoplasma gondii*, Rural landscape, Spatial distribution, Environmental contamination, Agent-based model

## Abstract

**Background:**

Predicting the spatial distribution of pathogens with an environmental stage is challenging because of the difficulty to detect them in environmental samples. Among these pathogens, the parasite *Toxoplasma gondii* is the causative agent of the zoonosis toxoplasmosis, which is responsible for public health issues. Oocysts of *T. gondii* are excreted by infected cats in the environment, where they may survive and remain infectious for intermediate hosts, specifically rodents, during months to years. The landscape structure that determines the density and distribution of cats may thus impact the spatial distribution of *T. gondii*. In this study, we investigated the influences of rural settings on the spatial distribution of oocysts in the soil.

**Method:**

We developed a spatially explicit agent based model to study how landscape structures impact on the spatial distribution of *T. gondii* prevalence in its rodent intermediate host as well as contamination in the environment. The rural landscape was characterized by the location of farm buildings, which provide shelters and resources for the cats. Specifically, we considered two configurations of farm buildings, i.e. inside and outside a village. Simulations of the first setting, with farm buildings inside the village, were validated using data from previous field studies. Then, simulation results of the two settings were compared to investigate the influences of the farm locations.

**Results:**

Model predictions showed a steeper relationship between distance to the nearest farm and infection levels when farm buildings, and thus cats, were concentrated in the same area than when the farms were spread over the area. The relationship between distance to the village center and level of environmental contamination also differed between settings with a potential increased risk for inhabitants when farms are located inside the village. Maps of the risk of soil contaminated with oocysts were also derived from the model.

**Conclusion:**

The agent-based model provides a useful tool to assess the risk of contamination by *T. gondii* oocysts at a local scale and determine the most at risk areas. Moreover it provides a basis to investigate the spatial dynamics of pathogens with an environmental stage.

**Electronic supplementary material:**

The online version of this article (doi:10.1186/1476-072X-13-45) contains supplementary material, which is available to authorized users.

## Introduction

Parasites with environmental stages are often challenging because of the difficulties to detect them in environmental samples [1, 2] and to predict their spatial distribution [3, 4]. The spatial distribution of the free living stages of such parasites may greatly depend on the behavior of their definitive hosts, which determines where the parasites are shed in the environment. The spatial distribution may also depend on environmental factors such as the site topography, the level of rain fall, and the temperature, which may influence transport and survival of these free living stages [5–8]. Many of these parasites are also zoonotic pathogens with an environmental stage that can directly infect animals and humans [9].

*Toxoplasma gondii* is a protozoa parasite responsible for the widely spread zoonosis toxoplasmosis, which can infect all warm blooded animals, including humans [10, 11]. Toxoplasmosis is largely known to cause severe symptoms in immuno-compromised persons or fetuses infected via congenital transmission [11]. Moreover, clinical cases were also reported in immuno-competent individuals [12–14]. Human infections result from the ingestion of parasite bradyzoites contained in contaminated meat, ingestion of oocysts from environmental sources, or congenital transmission [11]. Oocysts contribute to a large amount of infections in human and farm animals [15, 16]. It is thus necessary to understand their spatial distribution in the environment in order to evaluate the risk of infection from the environment. However, predicting oocyst spatial distribution in soil or water at a local scale remains difficult and poorly documented. In the present study we thus developed a spatially explicit model to investigate the distribution of *T. gondii* in rural environments.

Felids (wild and domestic cats) are definitive hosts of *T. gondii*. When infected, they can shed millions of oocysts in the environment via their feces. Hence, their defecation behavior may influence the spatial distribution of the parasite in the environment. However, this behavior is poorly documented. In urban environments, cats have small home ranges, and use localized common defecation sites [17, 18], whereas in rural environments cat feces may be dispersed over larger home ranges [19]. In rural environments, previous studies have underlined the role of farms, where cats concentrate at high density, on the level of environmental contamination: the probability of animal infection was found to be negatively related to the distance to the nearest farm [20, 21]. This spatial effect may result from farm locations, which were clustered and surrounded by fields, and from the behavior of cats that uses farms as shelters [21, 22]. However, the presence of oocysts in soil may not be limited to the immediate vicinity of households and farms: studies using intermediate hosts as markers of environmental contamination reported the presence of infected individuals in areas with low density of cats [23, 24] and contamination was still detected in 19.7% of soil samples located beyond 400 meters of the farm [25].

In order to predict the spatial pattern of environmental contamination by *T. gondii* oocysts in different rural settings, we developed an Agent Based Model (ABM) to take into account complex, heterogeneous and non-linear interactions between agents (cats and rodents), space and agents positions and behaviors [26]. Indeed, our model needed to integrate the landscape structure, the spatial contamination by the parasite and the behavior of rodents and cats. Traditional techniques such as non-linear dynamical systems (e.g. compartmental models) using differential equations would have lead to a very complex model using transition rates that aggregate several processes, while in the ABM, explicit rules govern the behavior of the different agents. Moreover, ABMs are very useful for modeling and visualizing processes at the spatial scale. Thus, the explicit description of individuals’ and environmental processes constitutes a more natural way of describing our system. We refer the readers to [27] for more detailed discussions on the merits of agent-based modeling of *T. gondii*.

A prototype ABM for the transmission dynamics of *T. gondii* was developed by Jiang et al. [27]. In their model, the authors explicitly described the transmission cycle of *T. gondii* in a farm and investigated the impact of oocyst survival and seasonality on cat and rodent seroprevalences. However, they did not address the question of the spatial distribution of *T. gondii* in the environment and hosts. Our model is an extension of that of Jiang et al. [27]. The study site is a 5.29 km^2^ area representing a village of North-Eastern France, also monitored for contamination of soil, cats and rodents [21, 25]. The model assumes that the location of cats depends on the size of their home range and farm buildings locations. As farm buildings concentrate cats, we expected the spatial distribution of the environmental contamination by *T. gondii* oocysts would depend on farm locations. Based on the same study area, we compared two settings of farm distribution: the first setting represents real locations of farm buildings concentrated at the periphery of a village, and the second setting consists in farm buildings spread over the area. For each setting, the levels of soil contamination and rodent infection were calculated as a function of the distance to the nearest farm and of the distance to the center of the village. The levels of infection according to the distance to the nearest farm represent the effect of the farm setting on the distribution of *T. gondii* in the environment and in rodents, while the levels of environmental contamination according to the distance to the center of the village provide an estimation of the risk of infection for people living in villages. The model was parameterized according to the knowledge and data available from previous field studies. For purpose of validation, results from the first setting were compared to field data obtained from the same site. Predictions of *T. gondii* spatial distribution from the two different settings were then compared to evaluate the impact of the spatial distribution of farm buildings.

## Materials and Methods

### Study site

The study site is an area of 5.29 km^2^ centered on the small village of Briquenay in northern France (49°24’19”N, 04°52’41”E). The landscape is heterogeneous with: a village at the center of the area, seven farms located at the edges of the village, and forests, crop fields and meadows in the periphery. The cat population of this site has been monitored since 2008, providing information on the serological status, cats age and population size.

In previous studies, we used direct measures to investigate the spatial distribution of *T. gondii* infection in intermediate hosts [21] and in soil [25]. Both of these contamination levels were best explained by the distance to the nearest farm. Results (field data and estimated probability of contamination) from these previous studies were then compared to the predictions of our model.

The agent based model, implemented in Netlogo 5.0.4 [28], was represented by a grid of 230 × 230 patches superimposed and centered on an aerial view (©IGN, 2010) of the village of Briquenay, with one patch representing 10 × 10 m^2^ (see Additional files 1 and 2: ODD protocol and NETLOGO script). In setting 1, actual farm building locations were used. In setting 2, the farm buildings were randomly spread over the area with a constraint of a minimal distance of 500 meters separating two farms, thus preventing farm clustering. In both settings, the center of the village was the geometric center of the modeled area.

### The agents of the ABM

We used two agent types in the model: cats (definitive hosts) and rodents (intermediate hosts). Both were autonomous and characterized by their own rules (see Additional file 1: Appendix ODD for the detailed rules). While rodents were initially spread randomly over the area, each cat was assigned to one of the seven farms. Cat home ranges were also assumed to be circular with a radius following a gamma distribution, Gamma (k,t), with k = 2 and t = 0.014 and centered on the cats’ farm [29, 30]. Cats’ locations were randomly chosen in the area delimited by their home range. Parameters and their sources are specified in Table 1.

**Table 1:** **Definitions and values of the main variables, functions and parameters in the model**

Process	Definition	Value	Ref.
	Number of X patches	230	
	Number of Y patches	230	
**Environment**			
Diffusion	Percentage of contamination diffusing to the 8 neighboring patches	10%	
Decay	Decontamination rate	1/125 day^−1^	[31]
**Rodents**			
Birth	Birth rate (*b*_*m*_)	10/365 day ^−1^	Value in the range of [27] and [32]
	Density dependent probability of birth of 1 juvenile by an adult rodent	*b*_*m*_ – 0.5(*b*_*m*_ –*m*_*m*_) × *N*_*m,i*_/*K*_*m,i*_	
Mortality	Mortality rate (*m*_*m*_)	2/365	[32]
	Density dependent probability of rodent mortality	*m*_*m*_ + 0.5(*b*_*m*_ –*m*_*m*_) × *N*_*m,i*_/*K*_*m,i*_	
Density	*N*_*m*,*i*_ = number of mice in the 46 × 46 patches (=460 × 460 m^2^) subdivision *i*		
	*K*_*m,i*_ **=** local carrying capacity of rodents (=Initial number of rodents) in the 46 × 46 patches subdivision *i*	300	*
Maturation	Weaning age of rodents	21 days	[27]
	Age at sexual maturation of rodents	50 days	[27]
Movement	Range of patches travelled (in any direction) randomly chosen	0-2 patches	[33]
Infection	Probability of vertical transmission	0.15	Value in the range of [27] and [32]
	Probability of rodent infection after ingesting one oocyst	1	[34]
	Number of contact between rodent and the patch/day	12	*
	Probability of infection from the environment while on patch *j*	1-(1-*p*_*conta, j*_)^12^	See above
	*p*_*conta, j*_ = level of contamination of patch *j*		
**Cats**			
Birth	Birth rate (*b*_*c*_)	2.4/365 day ^−1^	[32]
	Density dependent probability of birth of 1 juvenile by an adult cat	*b*_*c*_ – 0.5(*b*_*c*_ –*m*_*c*_) × *N*_*c*_/*K*_*c*_	
Mortality	Mortality rate (*m*_*c*_)	0.6/365 day^−1^	[32]
	Density dependent probability of cat mortality	*m*_*c*_ + 0.5(*b*_*c*_ –*m*_*c*_) × *N*_*c*_/*K*_*c*_	
Density	*N*_*c*_ = total number of cat on the entire site area		
	Carrying capacity of cats (=Initial number of cats)	100	order of magnitude observed in the field [19]
Maturation	Weaning age of cats	50 days	[27]
	Age at sexual maturation of cats	240 days	[27]
Movement	Random cat location per activity within their homerange		Estimated from maximum distance from [29, 30]
	Cat homerange radius picked in a gamma distribution	Γ(2, 0.014)	
Infection	Probability of predation of rodent within 1 patch (=10 m) of a cat	1	*
	Probability of cat infection after eating an infected rodent	1	[34]
	Probability of cat infection after ingesting one oocyst	10^−3^	[34]
	Probability of infection from the environment while on patch *j*	10^−3^ × *p*_*conta,j*_	see above
	Prepatent period before oocyst shedding	7 days	[10]
	Duration of infectiousness (=oocyst shedding period)	14 days	[10]
Environmental contamination	Probability of cat shedding oocysts per day	1	[35]
	Level of contamination on a patch *j* after shedding of oocyst by one infected cat	0.01 + *p*_*conta,j*_	See text

*chosen to obtain realistic values of rodent predation, rodent prevalence.

#### Population dynamics of the agents

Birth was a stochastic event depending on birth probability and density for both host populations (Table 1). Only mature individuals could reproduce. Cats were born in a farm randomly chosen, while rodents are born at the position of its parent. Mortality was also a stochastic event depending on the death probability and the population density (Table 1). For cat population, we used global density dependence, whereas local density dependence was used for rodent population. We used 460 × 460 m areas to implement local density dependence on rodent in order to maintain rodents within farm areas and to obtain a more homogeneous overall density of rodents than with global density dependence. Our choice for the size of these areas was based on the simulation time, which increased with smaller areas. Mice could also die because of predation by cats.

#### Agent daily activities

##### Cats

Each day, post-weaning cats could travel freely in one other single location within their home range, which results in a random position of the cat within its home range. Cats could get infected through contaminated environment according to their infection probability (Table 1) and the contamination level of the patch on which they were. They might also get infected by eating infected rodents. Cats were able to catch rodents within a radius of 10 meters of their position with a probability of success of 100% for each rodent, this probability was chosen *a posteriori* to obtain realistic predation rates.

Infected cats shed oocysts during two weeks after a prepatent period of 7 days [10]. Afterwards, we assumed cats acquired a lifelong immunity to the parasite and were thus detectable as seropositive to *T. gondii*[11]. We supposed that cats excrete one feces per day [19, 35].

##### Mice

Every day, mice travelled a random distance inferior to 20 meters which results in a daily random position of the mice within 20 meters of their previous location. Mice could get infected from contaminated patches with a probability depending on the level of contamination and the probability of infection after oocyst ingestion (Additional file 1: Appendix ODD protocol, Table 1). They might also get infected via vertical transmission with a probability of 15%. We assumed infected individuals remained infectious for cats and seropositive all their life.

### Environmental contamination dynamics

Patches were contaminated by feces of infected cats. We considered a level of contamination of 1% when an infected cat shed one feces on the patch. We assumed that one feces containing millions of oocysts would infect 100% an area of 1 m × 1 m, and thus 1% of a patch of 10 m × 10 m. Contaminated patches decontaminated over time at a rate 8.10^−3^ days^−1^, which lies in the range of decay rates of oocyst reported in [31]. To mimic the propagation of oocysts in the environment, caused by rainfall, animals, we assumed that patches diffused 10% of their contamination to their eight surrounding patches, every day. We assumed that patches with a contamination level above 5 × 10^−5^ are detectable by extraction methods [36].

### Variables reported for each simulation and analysis of the predictions

The model adopted discrete time in the step of one day. The simulation started at day zero and proceeded until day 3240. At day zero, all individuals and the environment were free of the parasite, excepted for 10 cats. This number was chosen in order to establish infection in all simulations because we focused on an area where *T. gondii* is endemic.

For each setting, we ran the model 200 times with each run representing 3240 days of simulation. This number and length of simulations allowed the stabilization of the average prevalences as a function of time (Additional file 3: Appendix A, Figure A-1 and A-2). We monthly tracked the age structure of the cat and rodent populations, as well as the number of infected and immune individuals in each age class and over time. The cat population was divided in six age classes: [0-1]; [1-2]; [2-3]; [3-4]; [4-5]; >5 years. The rodent population was split in five age classes: [0-2]; [2-4]; [4-6]; [6-12]; >12 months. These results are presented in graphical forms on supplementary data (Additional file 3: Appendix A).

We also monthly tracked the proportion of infected rodents, the proportion of contaminated patches (contamination level >5 × 10^−5^) and the average level of contamination in each of the following classes of distance to the nearest farm or of distance to the center of the village: [0- 100]; [100–200]; [200 - 300]; [300 - 400]; [400 - 500]; [500 - 600]; [600 - 700]; [700 - 800]; [800 - 900]; [900 - 1000]; >1000 meters.

For each run, the variables of interest were recorded and extracted from the software (netlogo). Results of simulations were imported into R [37] for analysis.

For the analyses of predictions, means and standard deviations of the different variables obtained at 3240 days over the 200 simulations were computed. We also compared simulation results of setting 1 with predicted values obtained from generalized linear modeling of field data [21, 25].

## Results

### Host population dynamics

We first checked that age structures of both host populations and prevalence in each age group after 3240 days of simulation were realistic for each setting (Additional file 3: Appendix A Figure A-3, A-4). We found a median age of cats < 1 year, which is consistent with other studies with median ages between 6 months and < 2 years [38–40]. Moreover, in a previous study located in another village in the same geographical area in France, the authors found a similar median age (4 months, [41]).

Cat population sizes and densities were 49.3 cats and 9.3 cats/km^2^ for setting 1 and 50.0 cats and 9.5 cats/km^2^ for setting 2, in the range of values reported in rural areas [19, 42, 43]. Cat predation rates were 36.8 rodents/cat/year (standard deviation (sd) = 17.1) for setting 1 and 45.7 rodents/cat/year (sd = 18.8) for setting 2, which are realistic values for predation in rural environment [44]. Illustrations of the outputs of the model for each setting are presented in Additional file 3: Appendix B.

### Overall seroprevalences in rodents and cats and global level of environmental contamination

The seroprevalences of both cats and rodents increased with age (Additional file 3: Appendix A Figure A-5 and A-6). The overall seroprevalences of the cat populations, predicted by the models, were 49.6% (sd = 8.3) with setting 1 and 50.9% (sd = 8.2) with setting 2, similar to seroprevalences reported in other rural sites of France (50.2% and 55.1% in [41], Table 2). In rodents, the overall seroprevalences were 2.0% (sd = 0.6) with setting 1 and 2.8% (sd = 0.8) in setting 2, slightly lower than the observed prevalence reported in the same site (4.0%) by [21]. The overall proportion of contaminated soil reached 11.2% in setting 1 (sd = 3.7) and 11.2% in setting 2 (sd = 3.9), while a proportion of 29.0% infected soil samples was reported on the same site [25] (Table 2). Cat, Rodent and environmental prevalences differed significantly between setting 1 and 2 (Table 2) with higher overall prevalences for setting 2.

**Table 2:** **Comparison of level of infection on cat and rodent populations and on environmental contamination**

Agents	Setting 1	Setting 2	Field
Cats	49.6%	50.9%^a^	Between 50.2% and 55.1% in other rural areas in France [41]
	Sd = 8.3	Sd = 8.2	
Rodents	2.0%	2.8%^b^	4,0% [21]
	Sd = 0.6	Sd = 0.8	
Soil	11.2%	11.2%^b^	29,0% [25]
	Sd = 3.7	Sd = 3.9	

^a^Values differs significantly between settings (glm, p =0.03).

^b^Values differs significantly between settings (glm, p <10^−3^).

### Presence of *T. gondii*in rodents as a function of distance to the nearest farm

**Figure 1:**
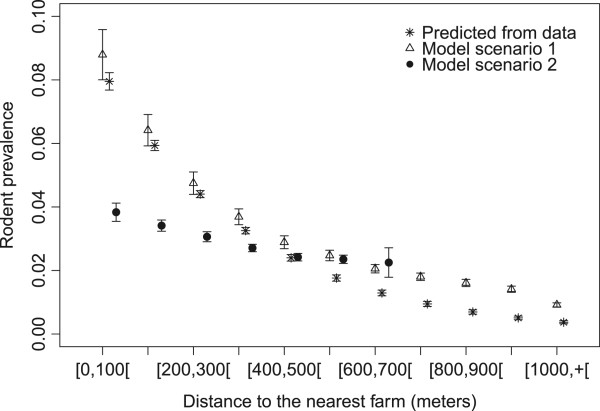
**Average seroprevalences in rodents for each class of distance to the nearest farm predicted by a logistic regression performed on field data probability of infection = f(distance to the nearest farm) (stars), by ABM model setting 1 with farm within the village (triangles), by ABM model setting 2 with scattered farms (filled dots).** 95% confidence intervals of the average of the 200 simulations (for each setting) were added for the ABM model predictions. The x-coordinates vary between the 3 data series for more readability of the confidence intervals.

Overall, predictions from setting 1 were in accordance with those from field data. Compared to setting 1, setting 2 predicted lower seroprevalences for the distance classes ranging between 0 to 400 m, while values predicted between 400 and 700 m values were similar to setting 1 (Figure 1). Note that there was no predicted value beyond 700 m for setting 2 because no patch was farther than 700 m of a farm. The distance to the nearest farm impacted more the rodent seroprevalence in setting 1 which was expected because of the concentration of farms in the village.

### Environmental contamination as a function of distance to the nearest farm

#### Detectable environmental contamination

The observed frequency of soil contamination on the study site was high (29.2%, [25]), with a higher frequency within core areas of cat home ranges (households and farms, which correspond to the first class of distance). The frequency decreased significantly with increasing distances from farms, but remained high (around 20%) at the periphery of the study site [25].

**Figure 2:**
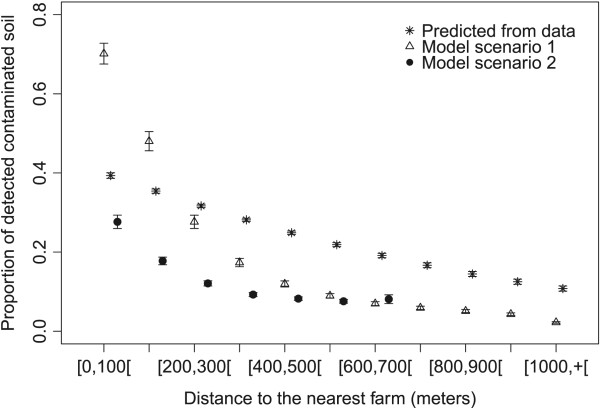
**Average proportion of detectable contaminated soil for each class of distance to the nearest farm predicted by a logistic regression performed on field data probability of contaminated sample = f(distance to the nearest farm) (stars), by ABM model setting 1 with farm within the village (triangles), by ABM model setting 2 with scattered farms (filled dots).** 95% confidence intervals of the average of the 200 simulations (for each setting) were added for the ABM model predictions. Note that there was no soil sampling further than 900 meters of any farm, and that in setting 2 there was no patch beyond 700 meters of any farm. The x-coordinates vary between the 3 data series for more readability of the confidence intervals.

Compared to field data, simulations from setting 1 predicted a higher frequency of detectable contamination near farms and lower frequency after 200 meters (Figure 2), thus the relationship between distance and frequency of contamination predicted by setting 1 was steeper than the observed one. Setting 2 predicted a lower detectable contamination than setting 1 for distance classes between 0–500 m. The decrease in contamination is also less marked than with setting 1. For both settings, between 600 and 700 meters, contamination levels were very similar (7.1% and 7.0% for setting 1 and 2 respectively).

#### Levels of contamination

**Figure 3:**
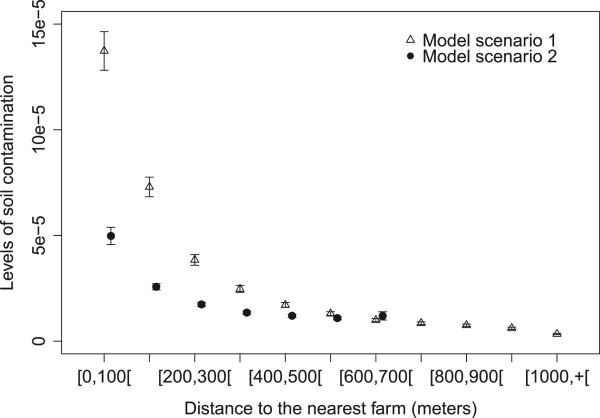
**Average levels of contamination for each class of distance to the center of the village predicted by ABM model setting 1 with farm within the village (triangles), by ABM model setting 2 with scattered farms (filled dots).** 95% confidence intervals of the average of the 200 simulations (for each setting) were represented. The x-coordinates vary between the 3 data series for more readability of the confidence intervals.

**Figure 4:**
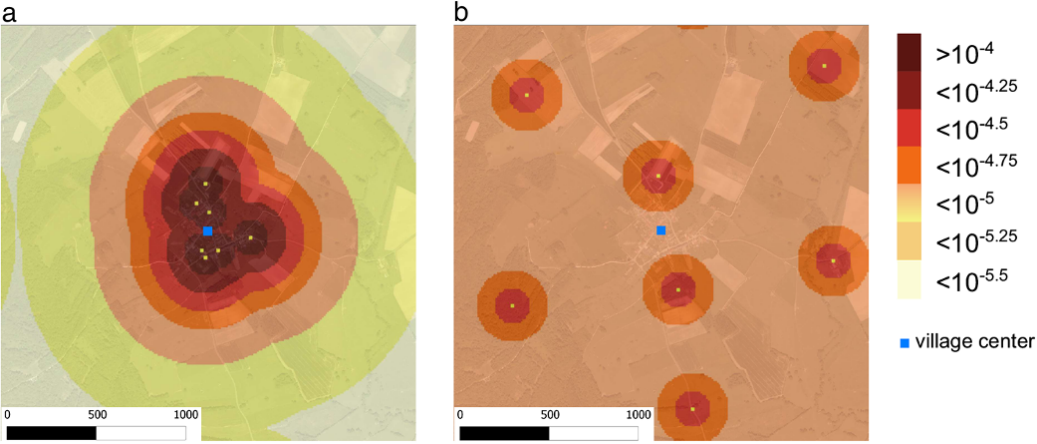
**Maps of the average level of environmental contamination predicted in the site by the ABM for each setting (** **a** **setting 1,** **b** **setting 2).** Farms are represented by yellow squares and the average levels of contamination of each distance class are represented by the different colors. Levels of contamination ranged from 10^-4^ to 10^-5.5^. The center of the village is for both settings the geometric center of the modeled area.

Field data could not provide the level of contamination of each sample, only the contaminated status of the sample was determined [25]. However the model provided the average level of contamination for each class of distance. For setting 1, contamination level decreased with increasing distance to the nearest farm, with the predicted level of the first distance class [0-100] being almost twice the level of the second class [100-200] (Figure 3). In setting 2, contamination levels decreased and were lower than from setting 1 from 0 to 400 meters from farms. Beyond this distance, few variations and no decrease were observed in the level of contamination (Figures 3 and 4b). The main differences between the two settings were lower contamination levels near farms (0 to 400 meters) and disappearance of the distance effect beyond 400 m in setting 2 compared to setting 1. However, in setting 1, a single area of high contamination was predicted in the center of the village, while setting 2 predicted several small areas of highest contamination (Figure 4a,b).

### Distribution of soil contamination as a function of the distance to the center of the village

The previous section compared the effect of the distance to the nearest farm on the spatial distribution of *T. gondii* in two settings of farm locations. In this section, we used the same simulations but compared the effect of the distance to the center of the village on the environmental contamination. This analysis provided estimations of the risk of environmental contamination and infection via oocysts for people living in villages with two different farm settings.

**Figure 5:**
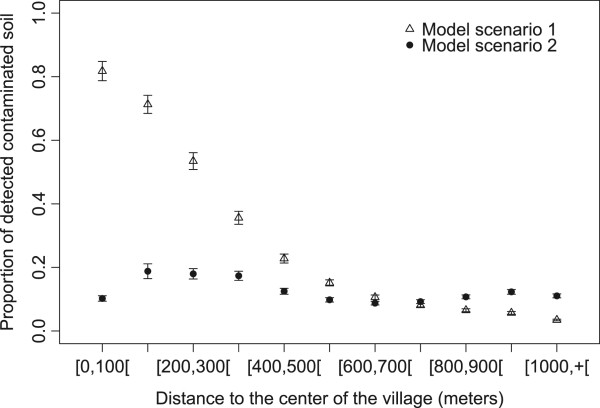
**Average proportion of detectable contaminated soil for each class of distance to the center of the village predicted by ABM model setting 1 with farm within the village (triangles), by ABM model setting 2 with scattered farms (filled dots).** 95% confidence intervals of the average of the 200 simulations (for each setting) were represented. The x-coordinates vary between the 3 data series for more readability of the confidence intervals.

**Figure 6:**
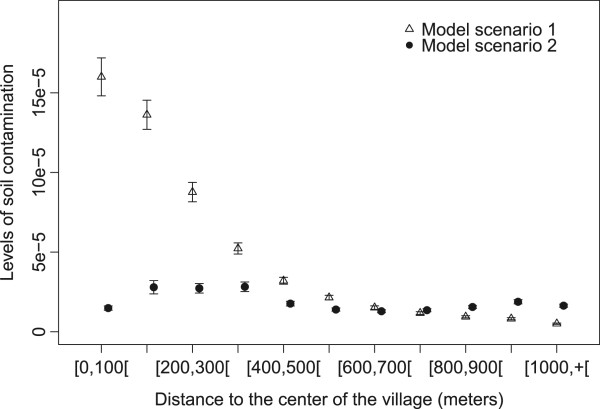
**Average levels of contamination for each class of distance to the center of the village predicted by ABM model setting 1 with farm within the village (triangles), by ABM model setting 2 with scattered farms (filled dots).** 95% confidence intervals of the average of the 200 simulations (for each setting) were represented. The x-coordinates vary between the 3 data series for more readability of the confidence intervals.

Different trends were observed between the two settings when the analysis is centered on the village (Figures 5 and 6). In setting 1, the risk of environmental contamination remained high up to 400 meters from the center of the village (81.8% to 35.6%) and decreased to 3.5% at 1000 meters and beyond. The risk of environmental contamination was lower in setting 2 up to 700 meters from the village center and was above setting 1 beyond this distance. No clear effect of the distance to the village center was visible in this setting. The pattern of soil contamination with distance to village center was very similar to the pattern observed with the distance to the nearest farm (Figure 2). Noticeably, in the first distance classes (0 to 300 meters), contamination levels in setting 2 were low compared to setting 1.

## Discussion

The model developed in this study provides a tool to study the environmental contamination and to build risk maps for parasite with an environmental stage. Overall, model predictions confirmed the importance of the farm buildings in concentrating environmental contamination and intermediate host infection by *T. gondii*[21, 25], thus increasing the risk of transmission to farm animals and humans living or working in farms. We also showed that the concentration of farms within the village increases the risk of soil infection in the village in comparison to a situation where farms are spread in the area. As a consequence, if the inhabitants mainly enter in contact with soil within the village, there might be a higher risk of contamination for them. On the contrary, when farms are outside the village, environmental contamination is evenly spread on the area and low in the center of the village. This may lead to lower risk for inhabitant of the village but could increase the risk for animals as seroprevalences in cats and rodents were slightly higher in setting 2.

### Comparison between predictions from setting 1 and field data

For cats, the probability of being seropositive to *T. gondii* is often reported to increase linearly with age [45, 46], which is in agreement with our predictions (Additional file 3: Appendix A2-A3). The predicted seroprevalences (48.8% and 51.9% for setting 1 and 2 respectively) are also similar to values reported in two rural sites in France (respectively 50.2% and 55.1%, [41], Table 2). For rodents, a positive relationship between rodent body mass, which is used as a proxy of age, and their probability to be seropositive for *T. gondii* has been found both within species [47] and between species [46, 48]. However, it seems that environmental factors could also largely influenced the probability of being positive for a rodent [21]. Considering the probability to be in contact with *T. gondii* increases during the life, the predicted trends seem coherent (Additional file 3: Appendix A4-A5). As well, the level of seroprevalence around 2% is also in agreement with observed prevalence in the site of Briquenay (4.0%) [21] (Table 2). The slightly lower prevalence could be attributed to the fact that we consider a larger area (5.9 km^2^) than in the field study (2.25 km^2^).

Predicted rodent prevalences from setting 1 agreed well with the spatial distribution of rodent infection observed in the field. However, the predicted levels of detectable soil contamination were lower than observed in the field. Several hypotheses could explain this difference. In the model, cats were considered as the main agent spreading oocysts. However transport and survival of oocysts may differ according to other environmental factors [5, 31], such as movements of agricultural engines and domestic animals, which are not included in the model. In addition, we assumed that cats have the same probability of contaminating any patch of their home ranges while they might favor some areas for hunting and defecating [29, 49]. More field data would be needed to include such factors. Model predictions showed a very similar spatial pattern for rodent infection and environmental contamination, which may be explained by the fact that in the model rodents were infected in majority by oocysts. The spatial pattern of rodent infection and environmental contamination were more different for the field studies. This might be explained by the fact that rodent and soil sampling were performed at different dates and the stochasticity of the infection/contamination events. Moreover, all rodent species do not have the same susceptibility to infection and all land use types are not favorable to the same species, which could contribute to differences in the shapes of spatial distribution of soil contamination and rodent infection. Nevertheless, model predictions confirmed the decrease of the presence of the parasite with increasing distance to the nearest farm, observed in the field, despite that the model predictions may overestimate its effect on the environmental contamination.

### Comparison between settings 1 and 2

#### Distance to the nearest farm

Spatial distributions of contaminated patches as well as levels of contamination showed similar patterns for both farm settings. For both settings, soil contamination was highest between 0 and 200 meters of the nearest farm. However, the levels of soil contamination in the vicinity of farm buildings were consistently at least twice higher in setting 1 than in setting 2. The concentration of farm buildings in the village led to a higher local density of cats in setting 1 compared to setting 2. The likelihood of soil contamination in the vicinity of farms is thus reduced in setting 2 compared to setting 1. Although contamination levels around farm buildings were low in setting 2 (Figure 2), this setting predicted a higher number of contamination hot spots across the site (Figure 4b).

#### Distance to the center of the village

Setting 1 predicted a high proportion of contaminated patches and level of contamination in the village, with the highest proportion in the first 0–100 meters of the center of the village. Both predictions showed that the contamination of the village was high when farms were aggregated within the village and at its periphery. On the contrary, when farms were scattered and not contiguous to the village (setting 2), soil contamination appeared to be much lower in the village center with little variation along the distance to the center. This was expected since, from the village perspective, farms buildings and thus cats, were spread over the area. However, it is worth mentioning that in the model we did not consider cats owned by inhabitants of the village. Although these cats may have smaller home ranges and lower predation rates on rodents than farm cats, they may still participate in the dissemination of *T. gondii* and thus increase the village contamination in both settings.

### Risk for human

Model predictions showed that the risk of exposure to oocysts for rural inhabitants or workers depends on the spatial distribution of farms. Concentration of farms in the same area increased the environmental contamination in this area. Thus, rural settings with isolated farm buildings may present lower risk of environmental infection both for farmers and villagers. In addition, in setting 2, configuration of farms may also limit infection risk of livestock grazing nearby farms, but increase the risk for livestock grazing farther away from the farms. In terms of public health, this study brings elements to assess the risk for people to get contaminated from contact with soil. These predictions should be coupled with information on the pattern of exposure of people. If we hypothesize that the contact of persons with soil is maximal within and around the village, decreasing the local density of cats may decrease the contamination in areas frequently used, and thus the risk for people. Moreover, these results may also be used to set up different degrees of monitoring of environmental contamination depending on village settings. For example, villages with a high density of farms would need a high level of vigilance in terms of infection risk of humans, while villages without farms or with scattered farms would require a lower degree of vigilance. This last hypothesis needs to be tested by field surveys comparing soil contamination in villages with different densities and farm settings.

### Limits of ABM model

A model has to be built at the right level of description for every phenomenon, judiciously using the right amount of detail for the model to serve its purpose [50]. Such factors like agent daily activities or environmental contamination dynamics are difficult to quantify, calibrate, and sometimes justify, which complicates the implementation and development of a model, as well as the interpretation of the simulation outputs. However, they were needed in the model as we wanted to predict the spatial contamination by *T. gondii* in two different settings considering the whole parasite transmission cycle. Another alternative to our ABM approach could have been to compute a risk map by integrating cat home ranges around farms and then delimiting spatial area at risk of *T. gondii* contamination using a Geographical Information System (GIS). However, this approach would not consider the interaction among agents and the dynamical processes such as population dynamics [51]. Here, we also aimed at providing a basic model that may be further investigated, thus modifying the population dynamics or agent behaviors may be needed and can be easily done with our model.

### Perspectives

The use of spatially explicit agent-based simulations opens interesting perspectives concerning the spatial dynamics of pathogen with an environmental stage or in predicting risk maps for humans. Several parameters were poorly known and chosen in order to obtain realistic values of prevalences. A sensitivity analysis would help targeting parameters that affect the most the results predictions, and thus evaluate the level of precision needed for the most unknown parameters. Implementation of field and experimental studies in order to obtain better estimation of these parameters would also greatly contribute to improve the accuracy of the model predictions. Additional variables could also be implemented, such as rainfall, temperature and seasonality of host population dynamics. These additional factors should provide a more complete spatio-temporal pattern of soil contamination for different seasons. Moreover, a comprehensive model taking into account these factors, as well as the presence of water sources will also contribute to predict transport of contamination potentially caused by water flows. Another possible extension is to include other intermediate host species to the model, such as farm animals and humans. The model may then be used to detail where and when the risk of infection occurs for each species, and thus help design prevention measures.

## Electronic supplementary material

Additional file 1: **ODD protocol of the ABM.** (DOCX 19 KB)

Additional file 2: **NLOGO script of the ABM.** (NLOGO 56 KB)

Additional file 3: **Agricultural landscape and spatial distribution of** ***Toxoplasma gondii*** **in rural environment: an agent-based model.** Appendix A: Age structures and seroprevalences in host populations. Appendix B: Illustrations of the model outputs. (DOCX 2 MB)
