# Supplementary material for: Agricultural landscape and spatial distribution of Toxoplasma gondii in rural environment: an agent-based model
Source: Int J Health Geogr. 2014 Oct 28;13:45. doi: 10.1186/1476-072X-13-45 (PMC4271439; doi:10.1186/1476-072X-13-45)
Supplement: Supplementary file 1 — Additional file 1: ODD protocol of the ABM. (DOCX 19 KB) [file 12942_2014_613_MOESM1_ESM.docx]

**Supplemental information: ODD protocol of the ABM**

Agricultural landscape and spatial distribution of *Toxoplasma gondii* in rural environment: an agent-based model.

**Purpose**

The purpose of this model is to predict the spatial distribution of the parasite *Toxoplasma gondii* in rodents and in the environment in two different rural scenarios: 1) the farms are concentrated at the edge of a village located in the center of the area; 2) the farms are spread over the area.

**Entities, State Variables, and Scales**

The model consists of 4 major entities: rodents, cats, farm buildings and environmental patches.

*Rodents*

Rodents are characterized by the state variables: age, infected, infection time, infection route (environment or vertical transmission), patch-ID, closest farm, and distance to the closest farm. Individuals which are less than 21 day old are referred as juveniles, between [21-50[ days are referred as sub-adult and all others as adults.

The rodent population is characterized by the number of rodents in the site area and the number of rodent in each subdivision of the area (used for implementation of a local density dependence on birth and mortality).

*Cats*

Cats are characterized by the following state variables: age, infected, infection time, immune, immune time, farm ID, infection route (predation or environment), home range radius, distance to their assigned farm, distance to their assigned farm when becoming infected. Individuals which are less than 50 day old are referred as juveniles, between [50-240[ days are referred as sub-adults and all others as adults.

The cat population is characterized by the number of cats in the site area.

*Farm buildings*

Farm buildings are static agents that contain state variables: coordinates and farm ID.

*Environmental patches*

The patches make up a 230x230 square grid landscape that represents roughly 5.29 km^2^. Each patch has state variables that include: contaminated, contamination time, contamination level, patch ID, closest farm, distance to the closest farm and distance to the center of the village.

**Process Overview and Scheduling**

The model proceeds in daily time steps. Within each time step, different phases are processed in the following order: increase age of rodents and cats; diffusion and decay of patch contamination; count number of rodent in each environment subdivision; rodents’ reproduction, death and activities; cats’ reproduction, death and activities. Infection and environmental contamination may occur during the rodents’ and cats’ activities. Vertical transmission in rodents may occur during reproduction.

**Design Concepts**

*Emergence*

The population dynamics of both host species emerge from the behavior of the individuals. The individual life cycle and behavior are represented by rules describing reproduction, mortality and activities which include infection. The parasite population is not explicitly represented but also emerges from the transmission cycle between cats, rodents and the environment. Finally the spatial distribution of the parasite appears from the relationships between the pathogen transmission cycle and the locations of the agents.

*Interaction*

Three different interactions are modelled. Birth and mortality of both host type are density dependent, birth decreases and mortality increases when the number of host is above their carrying capacity. When a rodent and a cat are close to each other, there is a probability that predation of the rodent occurs which can lead to cat infection if the rodent is infected and the cat is susceptible. When a rodent or cat is on a contaminated patch, there is also a probability that infection occurs if the host is susceptible.

*Stochasticity*

All demographic, behavioral and host infection parameters are interpreted as probabilities, or are drawn from empirical probability distributions. This was done to estimate the variations of the predictions. Two processes are deterministic: the decay and diffusion of oocyst, the reason is that we do not explicitely represent one oocyst but environmental contamination as a proportion or probability of contamination of a patch.

*Collectives*

Collectives are established through several agent states: Susceptible, infected, immune, age, distance to nearest farm, and farm ID. These states allow agents to be classified and are used to help determine subsets of agents that may provide useful information.

*Observation*

The data recorded by the model are the number of rodents and cats in each age class, the proportions of infected rodent per age class and class of distance to the nearest farm, the proportions of contaminated patches and the levels of patch contamination per class of distance to the nearest farm and to the center of the village. All of the recorded data is graphed in a “hidden” plot that can be exported and parsed using custom software in order to analyze the data.

**Initialization**

Population size of rodents and cats are set to their carrying capacity. *Toxoplasma gondii* is introduced by introducing 10 infected cats, this number ensure the spread of the parasite whereas introducing only 1 infected cat may lead to early extinction due to stochastic processes.
