# Supplementary material for: Agricultural landscape and spatial distribution of Toxoplasma gondii in rural environment: an agent-based model
Source: Int J Health Geogr. 2014 Oct 28;13:45. doi: 10.1186/1476-072X-13-45 (PMC4271439; doi:10.1186/1476-072X-13-45)
Supplement: Supplementary file 3 — Additional file 3: Agricultural landscape and spatial distribution of Toxoplasma gondii in rural environment: an agent-based model. Appendix A: Age structures and seroprevalences in host populations. Appendix B: Illustrations of the model outputs. (DOCX 2 MB) [file 12942_2014_613_MOESM3_ESM.docx]

**Supplemental Information**

**Agricultural landscape and spatial distribution of *Toxoplasma gondii* in rural environment: an agent-based model**

**Appendix A**: Age structures and seroprevalences in host populations

*Seroprevalence over time in the cat population*:

Figure A-1: Average cat seroprevalence of predicted by setting 1 (*a*) and by setting 2 (*b*) over time (in months). Bars represent standard deviations.

*Seroprevalence over time in the rodent population*:

Figure A-2 : Average rodent seroprevalence predicted by setting 1 (*a*) and by setting 2 (*b*) over time (in months). Bars represent standard deviations. Predicted seroprevalences are higher in scenario 2.

*Age structure of the cat population*:

Figure A-3: Average age structure of the cat population predicted by setting 1 (*a*) and by setting 2 (*b*). Standard deviations are represented. Age structures of the two cat populations are very similar.

*Age structure of the rodent population*:

Figure A-4: Average age structure of the rodent population predicted by setting 1 (*a*) and by setting 2 (*b*). Standard deviations are represented. Both age structures of the rodent populations are very similar.

*Age - seroprevalence relationship in the cat population*:

Figure A-5: Average cat seroprevalence in each age class predicted by setting 1 (*a*) and by setting 2 (*b*). Standard deviations are represented. The seroprevalence structured by age is similar in both scenarios with increasing prevalences with age.

*Age - seroprevalence relationship in rodents*:

Figure A-6: Average rodent seroprevalence in each age class predicted by setting 1 (*a*) and by setting 2 (*b*). Standard deviations are represented. The trend is similar for both scenarios however higher values are observed for scenario 2.

**Appendix B: Illustrations of the model outputs.**

Pink areas represent soil contaminated at a detectable level, with intensity of color proportional to the level of contamination, grey points represent rodents, blue, brown and green points represent cats, yellow squares represent farms.


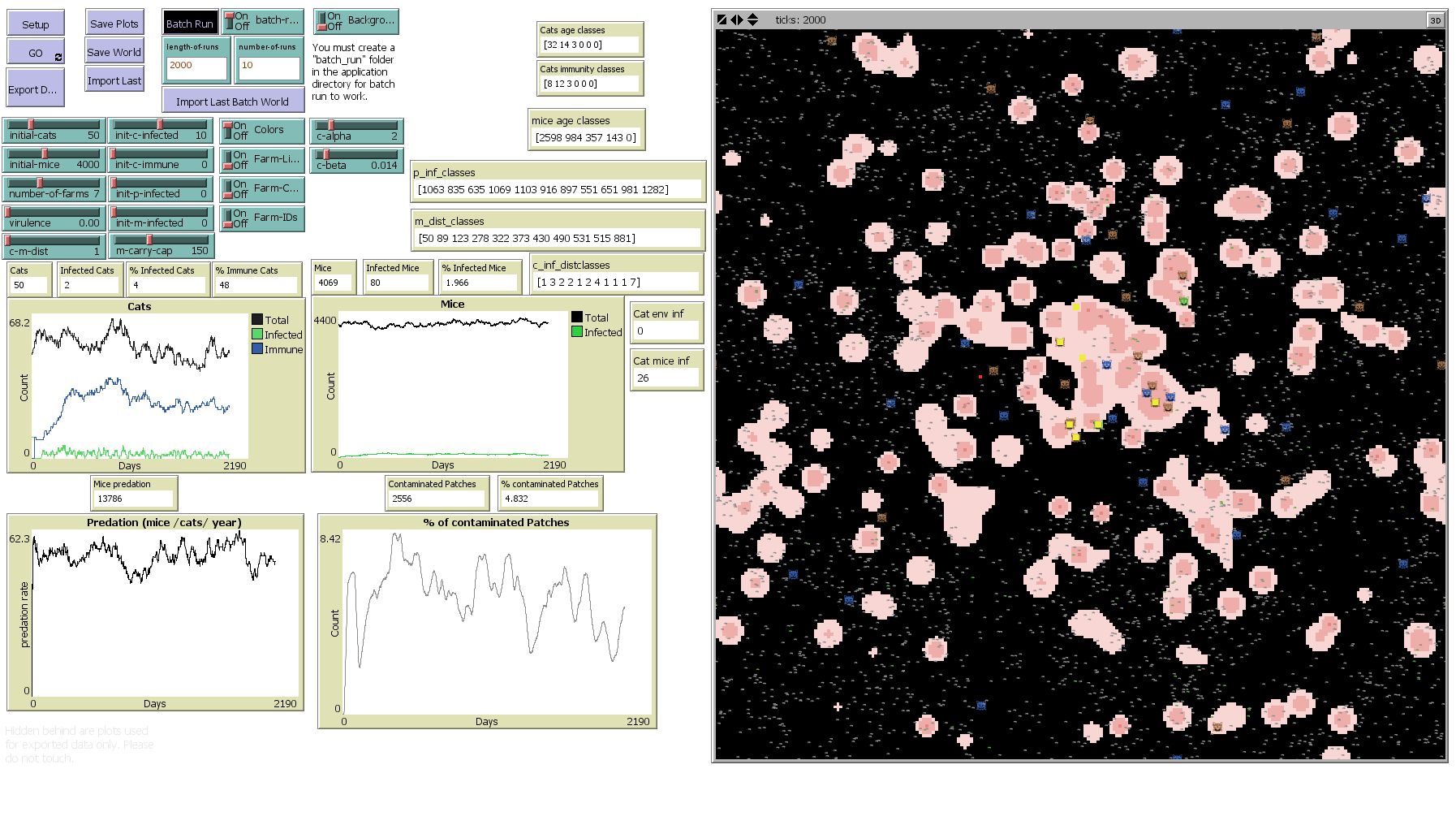

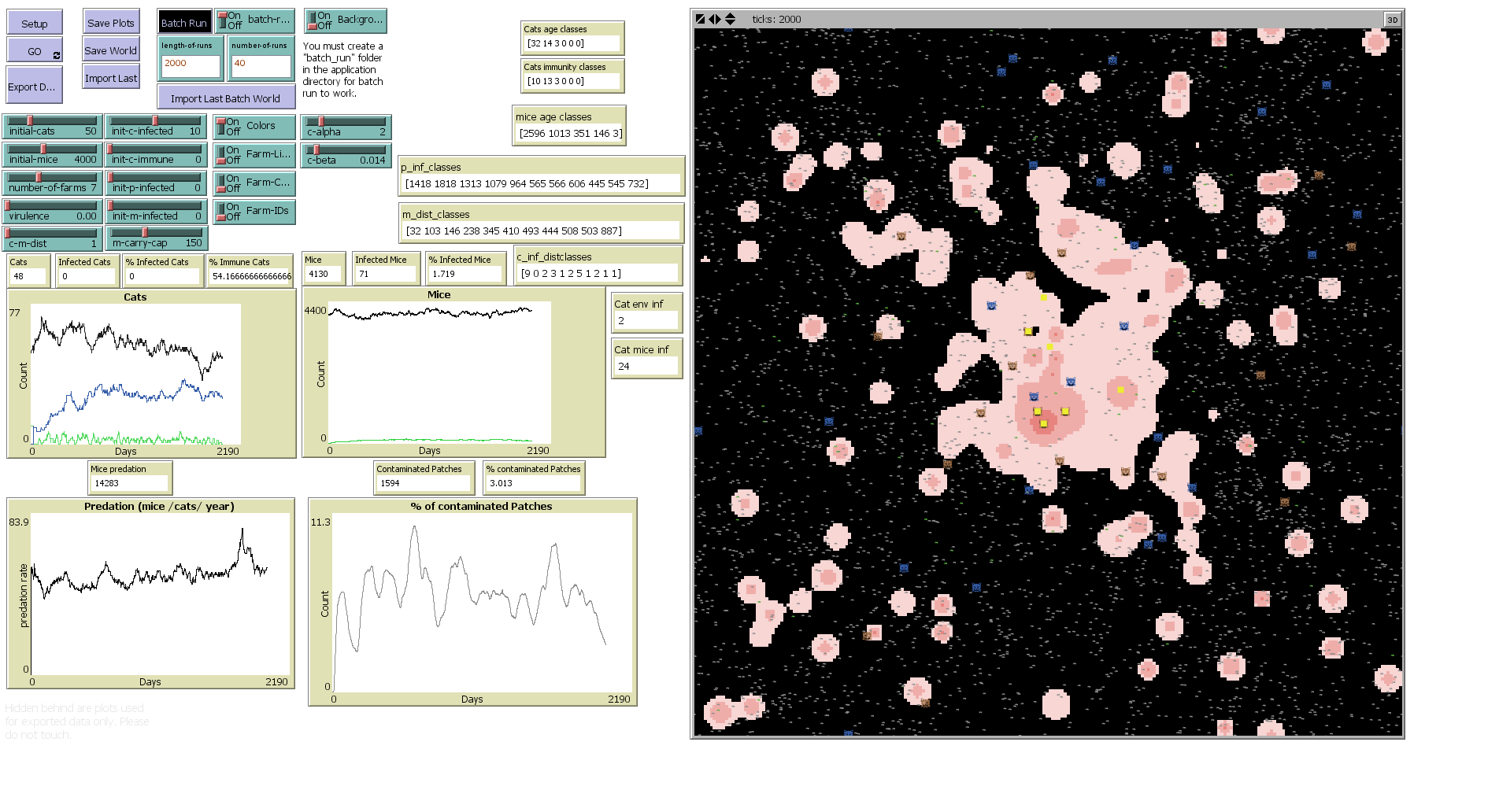


Figure B-1: Illustrations of two simulations of scenario 1 after 2000 days.


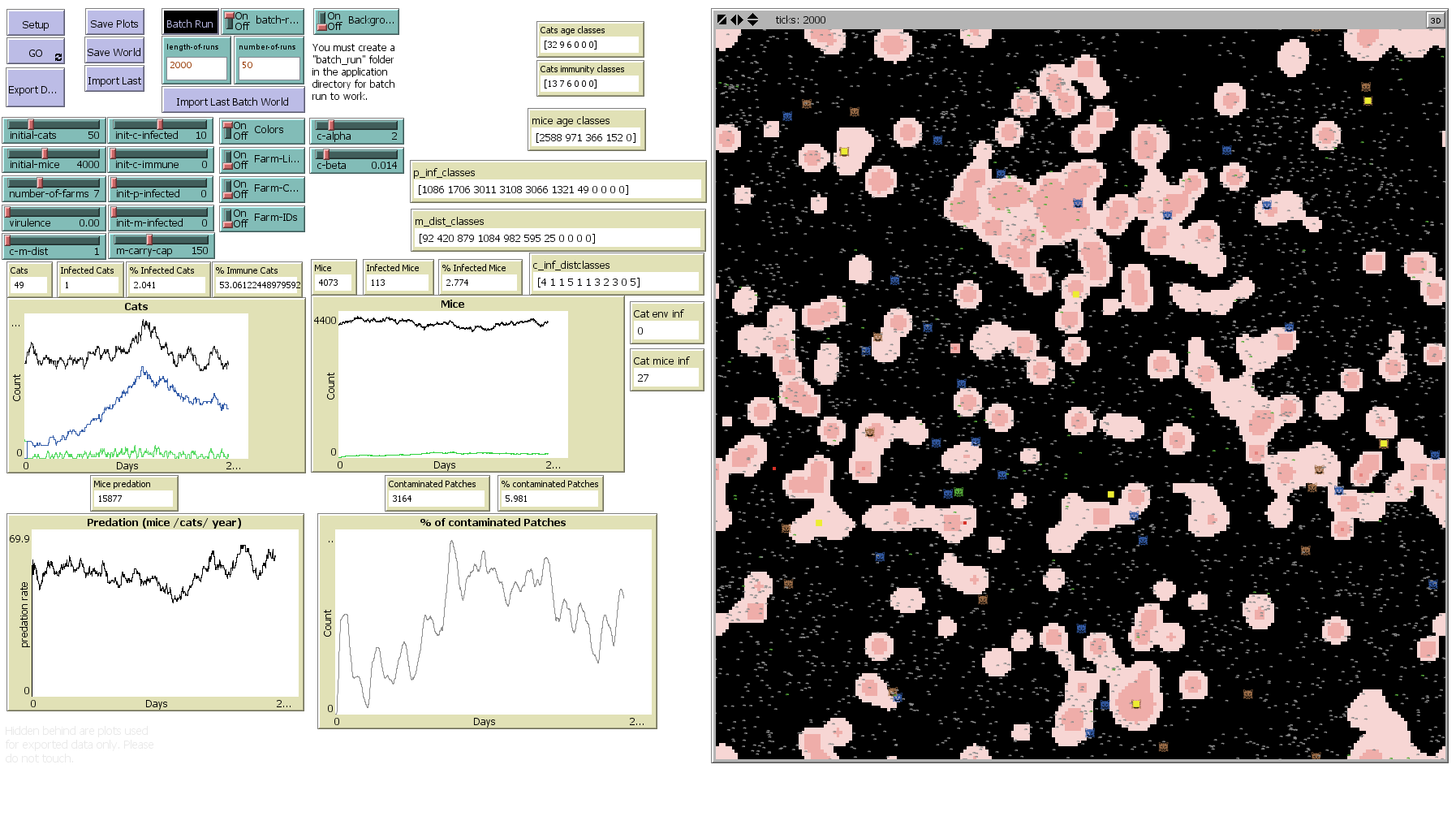

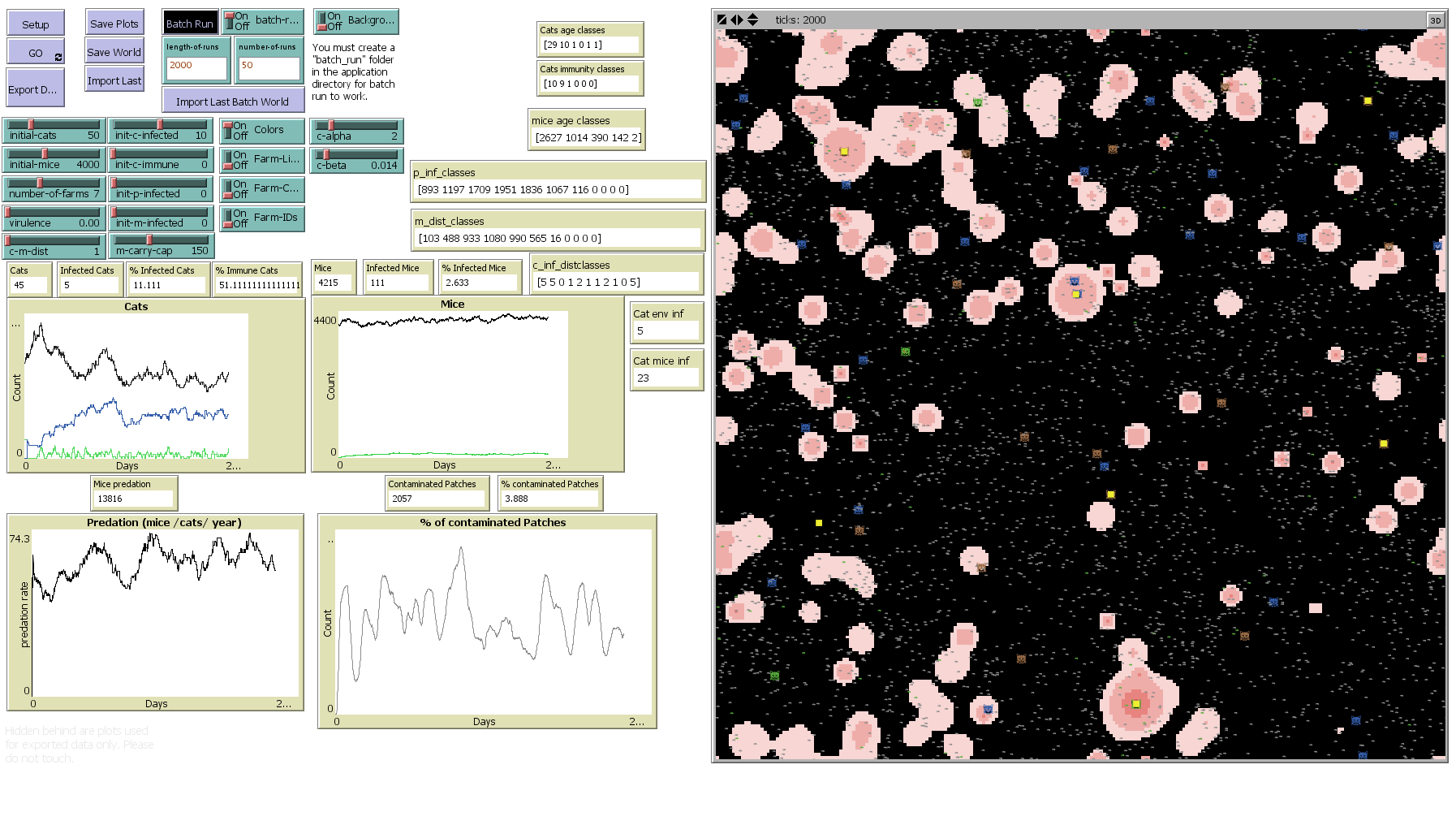


Figure B-2: Illustrations of two simulations of scenario 2 after 2000 days.
